# Schizophrenia: Redox Regulation and Volume Neurotransmission

**DOI:** 10.2174/157015911795596504

**Published:** 2011-06

**Authors:** I Bókkon, I Antal

**Affiliations:** 1 Doctoral School of Pharmaceutical and Pharmacological Sciences, Semmelweis University, Budapest, Hungary; 2 Department of Pharmaceutics, Semmelweis University, Budapest, Hungary

**Keywords:** Volume neurotransmission, redox regulations, dopamine, glutamate receptors.

## Abstract

Here, we show that volume neurotransmission and the redox property of dopamine, as well as redox-regulated processes at glutamate receptors, can contribute significantly to our understanding of schizophrenia. Namely, volume neurotransmission may play a key role in the development of dysconnectivity between brain regions in schizophrenic patients, which can cause abnormal modulation of NMDA-dependent synaptic plasticity and produce local paroxysms in deafferented neural areas. During synaptic transmission, neuroredox regulations have fundamental functions, which involve the excellent antioxidant properties and nonsynaptic neurotransmission of dopamine. It is possible that the effect of redox-linked volume neurotransmission (*diffusion*) of dopamine is not as exact as communication by the classical synaptic mechanism, so approaching the study of complex schizophrenic mechanisms from this perspective may be beneficial. However, knowledge of redox signal processes, including the sources and molecular targets of reactive species, is essential for understanding the physiological and pathophysiological signal pathways in cells and the brain, as well as for pharmacological design of various types of new drugs.

## INTRODUCTION

Converging evidence for the neurodevelopmental theory of schizophrenia suggests that a disturbance of brain development, involving genetic and environmental factors, during the intrauterine period and the first few years after birth underlies the later appearance of psychosis during adulthood [1]. However, the pathophysiology of schizophrenia is very complex and involves several cortical and subcortical systems. Brain imaging methods such as fMRI and PET have shown that differences linked to the neurocognitive deficits in schizophrenia most commonly occur in the frontal lobes, temporal lobes and hippocampus [2]. There have also been findings of differences in the structures and sizes of definite brain areas in schizophrenia.

The dopamine hypothesis of schizophrenia has been the major neurochemical assumption for over 40 years. This hypothesis is based on two fundamental observations: dopamine-enhancing drugs can produce psychosis, and blockade of D2 dopamine receptors is the sole property common to all antipsychotic drugs [3]. The dopamine hypothesis of schizophrenia proposes that excess activation of D_2_ receptors is the cause of the positive symptoms (*delusions, hallucinations, and thought disorder*) in schizophrenia. However, antipsychotic drugs that block dopamine D2 receptors are highly effective in treating the psychosis, but have limited effects on the negative symptoms (*deficits in social abilities and speech, affective flattening*).

Another hypothesis concerning the cause of schizophrenia postulates a deficit of glutamate functioning [4, 5]. Usually, it focuses on the glutamate neurotransmitter and the abnormally low level of the NMDA glutamate receptor in schizophrenia based on postmortem brains of people previously diagnosed with schizophrenia. The glutamate hypothesis is also supported by the fact that glutamate-blocking drugs such as ketamine and phencyclidine imitate the psychotic symptoms of schizophrenia.

Schizophrenia is also characterized by deficits of serotonin (5-HT) [6, 7] and norepinephrine [8]. Functional interactions between the central serotonin and dopamine (DA) systems have been well documented [9]. Alterations in the brain serotonin-dopamine balance are implicated in the etiology of schizophrenia. Numerous studies have shown that 5-HT receptors can modulate dopaminergic functions. However, the modulating effect of serotonin on striatal dopamine release is controversial. For instance, serotonin could inhibit [10, 11] or stimulate DA release in the striatum [12, 13]. The serotonin hypothesis is also supported by the known hallucinogenic effect of 5-HT receptor agonists, such as lysergic acid diethylamide (LSD), hallucinogenic derivatives of phenethylamine and tryptamine [14].

The hypofunction of subsets of GABAergic interneurons in the prefrontal cortex and the hippocampus was also suggested in schizophrenia [15]. The cholinergic system includes two families of receptors (*muscarinic cholinergic and the nicotinic cholinergic receptors*) that use acetylcholine as a neurotransmitter. Pharmacology, neuroimaging, and postmortem studies suggest alterations in the muscarinic cholinergic system in schizophrenia [16].

Mitochondrial dysfunction has also been proposed as a cause of schizophrenia, including dysfunction of the oxidative phosphorylation mechanisms, mitochondrial hypoplasia, and altered mitochondria-related gene expression [17]. There is also some evidence of irregular cellular metabolism and oxidative processes in the prefrontal cortex in schizophrenia, involving increased glucose demand and/or cellular hypoxia.

In addition, convergent evidence has implicated developmental dysregulation of glutathione (GSH) synthesis in the pathogenesis of schizophrenia [18-20]. Lack of regulation of neural calcium homeostasis has also been suggested as a link between the dopaminergic and glutamate abnormalities [21]. However, there is accumulating evidence for the presence of oxidative stress states (*dysregulated redox processes*) in diverse psychiatric disorders, which suggests that redox pathways present potential treatment targets that may be applicable to multiple disorders.

Here, we argue that the volume neurotransmission, antioxidant and pro-oxidant properties of dopamine, as well as redox processes at various glutamate receptors, may be essential to understanding schizophrenia. Fig. (**1**) demonstrates very complex features of several redox processes during activation of glutamate receptors.

## MONOAMINERGIC NEUROTRANSMISSION IS PREDOMINANTLY NONSYNAPTIC

Since Santiago Ramón y Cajal discovered gaps between neurons [22], neurotransmission has been considered to be a point-to-point synaptic connection. However, Paton and Vizi [23] and Knoll and Vizi [24] provided the first data indicating that neurons can communicate with each other without synapses. Vizi also provided evidence that this nonsynaptic neurotransmission (*volume neurotransmission, paracrine transmission, diffusion neurotransmission*) exists not only in the periphery but also in the brain [25, 26]. In the past few decades, several experiments have confirmed that neurotransmitters can be released from both synaptic and nonsynaptic sites. Thus, the extracellular space acts as an essential analog communication pathway in the brain. Volume transmissions have fundamental regulating roles in both presynaptic and postsynaptic processes.

The vast majority of monoaminergic varicosities create nonsynaptic contact that permit the release of transmitters directly into the extrasynaptic areas. It is estimated that about 95% of catecholamine (*the most abundant catecholamines are dopamine, norepinephrine, and epinephrine*) terminals are nonsynaptic and that each neuron is positioned within less than 30 µm of a norepinephrine bouton in the brain [27]. 

## REACTIVE SPECIES AS FUNDAMENTAL CELLULAR SIGNALS 

Thus far, reactive oxygen species (ROS) - hydrogen peroxide (H_2_O_2_), superoxide anion (O_2_^−**.**^), hydroxyl radical (HO^**.**^), and singlet oxygen (^1^O_2_) - and reactive nitrogen species (RNS) - nitric oxide (NO^**.**^) and peroxynitrite anion (ONOO^−^) - have been considered as very harmful agents that cause damage to macromolecules through nucleophilic attack [28]. These reactive species are generated primarily by the mitochondrial respiratory chain, NADPH oxidases, cyclooxygenases, lipoxygenases, cytochrome P450 oxidases, xanthine oxidase, nitric oxide synthase (NOS), etc. [29]. The harmful effects of reactive species are collectively termed oxidative stress and occur in biological systems when there is overproduction of ROS/RNS and/or a deficiency of enzymatic and non-enzymatic antioxidants [30, 31].

However, emerging evidence indicates that reactive species are essential signaling molecules for cells. Recent findings have shown that diverse kinds of reactive species and several products derived from their reactions, such as oxidized lipids and proteins, are necessary for regulating biological functions at all levels of biological organization [29, 32]. Reactive species and their derivatives stimulate distinct signaling pathways that can interact with each other. It may be paradoxical that diverse ROS-mediated processes protect cells against ROS-induced oxidative stress and restore redox homeostasis. During physiological (*pathophysiology*) processes, ROS and RNS can act as secondary messengers and control gene expression, apoptosis, cell growth, cell cycle, cell adhesion, chemotaxis, protein-protein interactions and enzymatic functions, Ca^2+^ and redox homeostasis, etc., in cells [29, 32-38].

## REACTIVE SPECIES AS FUNDAMENTAL SIGNALS IN THE BRAIN

During neuronal activity, a considerable volume of oxygen is used to maintain neuronal membrane potentials, which accordingly generate ROS and RNS. Various ROS and RNS as well as their derivatives act as signaling molecules in cerebral circulation and are required for signal processes such as synaptic plasticity and memory formation [39-41]. Reactive species control uptake of the excitatory neurotransmitter glutamate mainly by oxidation of protein sulfhydryl groups in rat cortical astrocytes [42].

The NO (*nitric oxide*) free radical molecule can freely cross cell membranes, acting as a neurotransmitter, neuromodulator, and messenger. Neuronal NO modulates synaptic activity by regulating neurotransmitter release and takes part in processes involved in synaptic plasticity, such as long-term potentiation, depression and synaptogenesis [43-46]. NO, as a diffusible messenger, increases glutamate release in NMDA receptor activation processes [47].

NADPH oxidases (*nicotinamide adenine dinucleotide phosphate-oxidase*) and other sources of superoxide are required for the hippocampal long-term potentiation (LTP) and hippocampus-dependent memory [48-51].

The NMDA subtype of glutamate receptors consists of a complex of various subunits. Since numerous cysteine residues are located in NMDA receptor subunits, which are redox-regulable, ROS and NO have important mediators of NMDA receptor signaling processes. During NMDA receptor activation, NADPH oxidase generates ROS *via* signaling pathway involving NO, cyclic guanosine monophosphate (cGMP), and protein kinase G (GPKG) [52].

Hydrogen peroxide (H_2_O_2_) is emerging as a ubiquitous messenger in various cells and the brain. Oxidative deamination of monoamines by mitochondrial MAO is accompanied by the reduction of molecular oxygen to H_2_O_2_. Recently, Bao *et al.*, [53] demonstrated that respiring mitochondria are the primary source of H_2_O_2_ generation for dynamic neuronal signaling.

In brief, many experiments have provided evidence that reactive species act as essential signals during physiological (*pathophysiological*) processes in cells and the brain.

## MITOCHONDRIAL NETWORKS AS REDOX CENTERS AND METABOLIC HUBS 

To date, usually, mitochondria are considered as simple cellular energy sources in most scientific literatures. However, there is accumulating evidence that mitochondria function as essential centres for cellular signaling pathways in cells. According to Aon *et al.*, [54, 55] mitochondria can function as metabolic hubs that produce ROS signaling molecules with scale-free dynamics. Namely, the coordination between mitochondria within the network appears to be ROS mediated. Mitochondria are poised at the convergence of most anabolic and catabolic pathways through the tricarboxylic acid cycle. Thus, mitochondria can act as metabolic and redox hubs due to their numerous links to other pathways as inputs (*sources*) or outputs (*sinks*). In addition, growing evidence demonstrates that shapes and spatiotemporal arrangements of mitochondria can be very different in different cell types [56]. Moreover, activity-dependent mitochondrial redistribution takes place in neurons [57].

Mitochondria are essential determinant of the excitability and viability of neurons. Mitochondria take up about 25% of the cell volume in neurons and play fundamental roles in cellular redox and Ca^2+^ homeostasis, ATP generation, free radical production, regulation of neurotransmitter release, cell growth, apoptosis, cell signaling, iron metabolism, steroidogenesis, and many other functions [40, 58].

In contrast to the textbook description of mitochondria as small spherical organelles in cells, mitochondria in muscular, neuronal and connective tissue are principally filamentous [56, 59, 60]. Mitochondria are functionally connected, i.e., they produce a dynamic network within neuronal cells. Mitochondria continuously fuse and divide, and their morphology and intracellular distribution change according to the energy demands of cells [61, 62]. Because mitochondria are key players in cellular redox homeostasis and signaling and one of the main sources of free radicals, they play a central role in redox-dependent post-translational reversible oxidative modifications of proteins such as tyrosine phosphatases and protein tyrosine kinases.

Mitochondria also participate in the synthesis and secretion of neurotransmitters. Some essential steps in the metabolism of the major excitatory neurotransmitter glutamate and the principal inhibitory neurotransmitter GABA (*gamma-aminobutyric acid*) take place in the mitochondrial tricarboxylic acid cycle [63].

Besides, mitochondrial monoamine oxidase (MAO) enzyme performs a key metabolic role in the turnover of serotonin, dopamine, norepinephrine, and epinephrine in the brain [64-66]. Oxidative deamination of monoamines by mitochondrial MAOs is accompanied by the reduction of molecular oxygen to H_2_O_2_. Namely, reactive species are generated by mitochondrial monoamine oxidases during natural metabolism of serotonin, norepinephrine, epinephrine, and dopamine. Because oxidative deamination of monoamines by mitochondrial MAOs is a regulated process, reactive species generated during oxidative deaminations may also serve as essential signaling molecules in cells.

In contrast, excess (*unregulated*) production of dopamine (*monoamines*) can induce overproduction of H_2_O_2_ and superoxides *via* monoamine oxidases, as well as leading to an excess of auto-oxidized dopamine that can cause lipid peroxidation and DNA and protein modifications and interact with the mitochondrial electron transport system. Dopamine can inhibit brain mitochondrial respiration that involves generation of reactive oxygen species [67]. In addition, dopamine-associated inhibition of mitochondrial respiration is dependent on MAO and H_2_O_2_ [68]. 

## DOPAMINE AND SEROTONIN CAN REVERSIBLY REGULATE MITOCHONDRIAL MOTILITY AND DISTRIBUTION

Recently, it was demonstrated that the neurotransmitter dopamine and serotonin (5-HT) can reversibly regulate mitochondrial motility and distribution in cultured hippocampal neurons [69, 70]. Chen *et al.*, found that dopamine bears a net inhibitory effect on mitochondrial movement. In contrast, 5-HT performed a stimulatory effect on mitochondrial movement. Chen *et al.* suggested that dopamine and 5-HT can determine the distribution of mitochondria as energy sources in neurons.

However, because mitochondria can function as metabolic and redox hubs, the distribution of mitochondria represents a distribution not only of energy sources but also of metabolic and redox processes. In other words, when dopamine and 5-HT determine the distribution of mitochondria in neurons, they also determine cellular redox and Ca^2+ ^patterns, ATP generation patterns, free radical patterns, spatiotemporal patterns of various cell signals, neuronal membrane potentials, and many other parameters. It is very possible that other neurotransmitters, such as norepinephrine and acetylcholine, can also affect mitochondrial movement and distribution.

## CATECHOLAMINES AND SEROTONIN AS ANTIOXIDANTS

Catecholamines and serotonin and their metabolic products can be either neurotoxic or neuroprotective. However, catecholamines and serotonin bear free radical scavenging and neuroprotective abilities [71-74]. At high doses, catecholamines induce apoptosis but prevent free radical-mediated neurotoxicity as antioxidants without being coupled to the receptors [74]. The catechol structure is a fundamental component for the antioxidative effect of catecholamines. The redox state of the cell is largely linked to the iron (*and copper*) redox couple and is maintained within strict physiological limits. Catecholamines can inhibit generation of free radicals by chelating various metals; i.e., they can balance redox potential by complex formation [75, 76].

Serotonin and its precursor have great antioxidant properties in the brain [73]. Serotonin attenuates free radical-induced neuronal death without being coupled to serotonin receptors in cultured mouse cortical neurons [77]. Norepinephrine reduces caspase activation and ROS production in cholinergic neurons [78].

Dopamine and its five receptor subtypes play various roles in the central nervous system. Dopamine exerts its actions through two families of cell surface receptors that belong to the class of G protein-coupled receptors. D1-like receptors (D1, D5) stimulate adenylyl cyclases, while D2-like receptors (D2, D3, D4) inhibit adenylyl cyclases [79]. Activation of D1 and D5 receptors produces antioxidant responses [80, 81]. Antioxidant response to the D5 induction is mediated by inhibition of NADPH oxidase activity [80]. Besides, dopamine has concentration-dependent effects on ROS production. It acts as an antioxidant at physiological concentrations, but as a prooxidant at higher concentrations. The neuroprotective outcomes of dopamine can be both receptor-mediated and non-receptor-mediated [82]. However, dopamine is a potent antioxidant, and when it reduces reactive oxygen species, it is converted into neurotoxic dopamine *o*quinones. Dopamine *o*quinone can rapidly recover to dopamine by an ambient antioxidant such as glutathione or ascorbate. In the absence of ambient antioxidants, *o*quinones form neurotoxic *o*semiquinones, which are free radicals [83].

Because mitochondria play key roles in the synthesis and secretion of classical neurotransmitters and that catecholamines and serotonin can act as antioxidants and free radical scavengers, mitochondria can also control redox processes *via* the regulation of neurotransmitter metabolism and secretion.

## GLUTATHIONE IN SCHIZOPHRENIA

The cellular thiol redox state is a fundamental mediator of numerous signaling, metabolic, and transcriptional processes in cells. The glutathione (GSH) and thioredoxin (TRX) systems are the two key and ubiquitously expressed antioxidant systems that reduce thiol (-SH) groups [84]. The GSH and TRX systems maintain a reduced intracellular redox condition in cells by the reduction of protein thiol groups. Namely, they keep signaling components in a reduced state and are counterbalanced in signaling by oxidative mechanisms. GSH (*g-glutamyl-cysteine-glycine, a tripeptide that exists in reduced monomeric (GSH) and oxidized dimeric forms* (GSSG)) is the major thiol antioxidant and redox buffer in cells and is abundant in the cytosol, nuclei, and mitochondria.

There is increasing evidence that the glutathione metabolism is abnormal in schizophrenia and that a weakened capacity to synthesize GSH under oxidative stress is a susceptibility factor for schizophrenia. Namely, patients with schizophrenia indicate a deficit in glutathione levels in the prefrontal cortex and cerebrospinal fluid, as well as the reduction of gene expression of GSH-synthesizing enzymes [18, 19, 85]. However, glutathione can act as a neuromodulator at the glutamate receptors and as a neurotransmitter at its own receptors. GSH plays a major role in modulating redox-sensitive sites, including NMDA receptors [86]. In slices of rat hippocampus, reduced GSH levels weaken NMDA-mediated responses and synaptic plasticity [20].

Cabungcal *et al.* [87] provide experimental evidence that glutathione deficit, during postnatal development, induces dysfunctions in GABAergic neurons in anterior cingulate cortex of rats. These dysfunctions of GABAergic interneuron can have wide-ranging effects on the neuronal circuitry of prefrontal cortex and its output to additional brain areas.

## REDOX REGULATION AT IONOTROPIC NMDA RECEPTORS

Glutamate activates two types of glutamate receptors: ionotropic (iGluRs) and metabotropic glutamate receptors (mGluRs) [88]. Ionotropic glutamate receptors have been subdivided into α-amino-3-hydroxy-5- methylisoxazole-4-propionic acid (AMPA), kainite, and *N*-methyl-D-aspartate (NMDA) receptor subclasses.

It is accepted that the glutamate ionotropic NMDA receptor is the principal molecular structure for controlling synaptic plasticity and memory function. NMDAR activation starts several events, including calcium influx, activation of nitric oxide synthase, and superoxide formation [89, 90]. Earlier studies have suggested that mitochondria are a principal source of NMDA-induced superoxide production. However, recent experiments have indicated that NADPH oxidase can be the main source of superoxide production following neuronal NMDAR activation [91]. It is probable that initial superoxide signals produced by NADPH oxidase can stimulate secondary mitochondrial superoxide production.

Redox modulation has been recognized as an essential system in the regulation of the NMDA receptor [86]. Oxidizing agents decrease, but reducing agents enhance NMDA-evoked currents. Numerous cysteine residues are located in NMDA receptor subunits, which are redox-regulable.

It is possible that redox agents can produce conformational changes in the NMDAR protein at cysteine residues to promote or inhibit the development of disulfide bridges, which induce changes in channel properties [92]. The NMDA receptor can also be regulated by nitric oxide (NO)-related species (*not involving cyclic GMP*) and endogenous glutathione [86].

During excitation of a neuron by glutamate, nitric oxide is produced that stimulates exocytosis and release of dopamine and glutamate from nearby neurons [93]. Thus, dopamine can alter the strength of glutamatergic synapses and affect synaptic plasticity. It is possible that nonsynaptic volume transmission of dopamine (*as a form of spatiotemporal signaling*) complements classic synaptic transmission and organizes local activity among neuron groups.

Smythies has presented research about the redox biochemical basis of learning and neurocomputation [94-97]. His theory is based on redox mechanisms at the glutamate synapse. He emphasized that catecholamines are potent anti-oxidants and free radical scavengers. According to Smythies [94], “…it is possible that catecholamines from the *en passage *DA bouton (and possibly norepinephrine (NE) boutons in NE systems) may modulate the redox status of the adjacent glutamatergic synapse by scavenging ROS”. However, Smythies was the first who emphasized that synaptic and nonsynaptic (*volume transmission*) transmission processes can be redox-based mechanisms.

## IONOTROPIC AMPA GLUTAMATE RECEPTORS AND REGULATION OF STRIATAL DOPAMINE RELEASE BY DIFFUSIBLE H_2_O_2_

AMPA (*α-amino-3-hydroxy-5-methyl-4-isoxazolepropionic acid*) ionotropic glutamate receptors are expressed in every part of the mammalian brain and are composed of various combinations of glutamate receptor subunits (GluR1–GluR4) [98, 99]. Glutamate receptor subclasses can coexist in the same cell [100]. Subunits and splice variants of AMPA receptors also differ by brain region.

Regulation of dopamine-dependent glutamate transmission is relatively known. For instance, glutamate release can be inhibited by D2 dopamine receptors on corticostriatal afferents [101]. This dopamine regulation is achieved by nonsynaptic volume neurotransmission [102].

In contrast, how synaptically released glutamate control dopamine release in striatum is less understandable. However, the effect of ionotropic glutamate-receptor activation on dopamine release should be indirect because AMPA receptors are not expressed on DA terminals [103, 104]. Several experiments support that H_2_O_2_ can function as a signaling messenger in the brain and other tissues by various H_2_O_2_ regulable processes such as kinases, phosphatases, transcription factors, ion channels, etc. [32, 105-107].

Recently, Avshalumov *et al.* [108] reported that activation of ionotropic AMPAR produce diffusible H_2_O_2_, which opens ATP-sensitive potassium (K_ATP_) channels and thereby inhibits synaptic dopamine release in striatum (the major input station of the basal ganglia system). In other words, the mechanism by which H_2_O_2_ can inhibit dopamine release is activation of ATP-sensitive potassium channels.

The major subcellular sources of H_2_O_2_ production are mitochondrial respiration, monoamine oxidase (MAO), and NADPH oxidase. Very freshly, Bao *et al.* [53] demonstrated that fast H_2_O_2_ production is due to the mitochondrial respiration during glutamatergic activation of AMPA receptors in the striatum. Namely, respiring mitochondria are the major sources of H_2_O_2_ generation for fast, dynamic neuronal signaling. In contrast, MAO and NADPH oxidase do not contribute to dynamic, fast (*subsecond*) H_2_O_2_ signaling in the striatum, nevertheless these act as H_2_O_2 _sources at longer time scales.

## REDOX-CONTROLLED PHOSPHORYLATION OF NMDAR SUBUNITS 

The phosphorylation of NMDAR subunits by protein kinases plays a critical role in NMDAR regulation and synaptic plasticity [109, 110]. For instance, NMDAR currents can be enhanced by increasing tyrosine kinase activity [110].

Numerous studies have demonstrated that reversible oxidative modifications (*reversible thiol modification*) of protein tyrosine kinases and protein tyrosine phosphatases by ROS play essential roles in regulating their enzymatic activity [111-113]. Although these oxidative modifications are reversible, their effects on the enzymatic activity of tyrosine phosphatases and tyrosine kinases can be even opposite. Thus, protein tyrosine phosphorylation-based signaling pathways are under the regulation of reactive oxygen species.

Li *et al.*, suggested, [114] that an impairment in NR1 subunit phosphorylation of NMDA receptors produces glutamatergic hypofunction that can contribute to behavioral deficits related to psychiatric disorders. However, these findings indicate that phosphorylation of NMDAR subunits by protein kinases is also subject to regulation by redox and free radicals.

## METABOTROPIC GLUTAMATE RECEPTORS

The metabotropic glutamate receptors (mGluRs) are G-protein-coupled receptors with seven transmembrane domains that can modulate brain excitability *via* presynaptic, postsynaptic and glial mechanisms To date, the existence of eight metabotropic glutamate receptors (*from mGlu1 to mGlu8*) have been identified, and these receptors have been clustered into three groups (I-III). mGluRs are not ion channels but are active through an indirect metabotropic process [115]. mGluRs modulate the activity of ligand- and voltage-gated ion channels by means of G-protein-mediated activation of such intracellular messenger cascades as phospholipase C, adenylyl cyclase and protein kinase C.

In contrast to the glutamate ionotropic receptors, such as NMDA, AMPA and kainate receptors, which are responsible for fast excitatory transmission, mGluRs play modulatory roles in glutamatergic synaptic transmission by modulating the ion channel activity and neurotransmitter release. mGlu receptors can regulate neuronal function separately from the ionotropic NMDA receptors (iGluRs) and protect against cellular injury by regulating oxidative and redox processes. For instance, glutamate can induce dopamine efflux by metabotropic GluR1 activation.

However, mGluR agonists can modulate - increase or decrease - the release of dopamine in the striatum and nucleus accumbens [116-118]. One possible explanation for this contradiction is that mGluR subtypes differently modulate the release of extracellular dopamine in different areas of the brain [117]. It was suggested that mGluRs can regulate neuronal injury and survival, likely *via* protein kinases and cysteine protease signaling pathways that affect mitochondrial controlled programmed cell death [119].

However, since mGluRs can modulate the release of dopamine (dopamine can acts as an antioxidant at physiological concentrations and modulate the redox status of the adjacent glutamatergic synapse), and act *via* protein kinases and cysteine protease (ROS play essential roles in regulating their enzymatic activity) signaling pathways that affect mitochondrial processes (mitochondrial networks are the main sources of ROS), indicating that mGluR associated signal pathways are also redox-linked processes.

## MODULATION OF NMDA RECEPTORS AND DOPAMINE TRANSPORTERS BY ZINC 

Functional native NMDAR, which is a heteromultimer of NR1 and NR2 subunits [120, 121], play multifaceted roles in synaptic plasticity. Zinc can act *via* these receptors to modulate bidirectional plasticity. The existence of zinc in synaptic terminals and its extracellular release during synaptic transmission [122, 123] suggest that zinc is a significant modulator of synaptic plasticity. For instance, zinc can modulate synaptic plasticity in hippocampal CA1 region by different mechanisms that are dependent on zinc concentrations [124].

The dopamine transporter (DAT) is a specific membrane protein in dopaminergic neurons. Dopamine reuptake by DAT regulates the extracellular dopamine concentration in brain areas with dopaminergic innervation. Lately, Pifl *et al.* [125] demonstrated the first evidence that DAT regulation by Zn^2+^ is intensely modulated by the membrane potential and chloride. In other words, the direction (*inhibitory or stimulatory*) of the Zn^2+^ effect on dopamine reuptake depends on the membrane potential and chloride distribution of cells.

Intra- and extracellular redox states are closely linked to various ions. During synaptic transmission, release of zinc ions into extracellular space serves an important modulatory role in plasticity. The catechol structure is an essential component for the antioxidative effect of dopamine. However, dopamine may also play a functional role in the regulation of zinc ions by chelating them, which can in turn have an indirect modulatory effect on NMDAR function and a modulatory effect on dopamine reuptake. In fact, some studies indicate that extracellular zinc chelators modulate NMDAR in the CA3 region [126].

## REDOX-RELATED EPIGENETICS IN SCHIZOPHRENIA

Recent works indicate that an epigenetic mechanism is also an attractive hypothesis for a molecular contribution to schizophrenia. Accumulating evidence shows that schizophrenia may arise from the abnormal epigenetic regulation of multiple genes. CpG islands (*made up of CpG dinucleotides clustered together*) play a critical role in controlling gene expression within the promoters of specific genes. Cytosine methylation (*i.e., formation of 5-methylcytosine, catalyzed by a DNA cytosine-5 methyltransferase (DNMT*)) is found essentially at CpG dinucleotides in animals [127, 128]. In general, the epigenetic silencing of gene expression is related to the hypermethylation of CpG islands. It has been suggested that abnormal gene expression (*for instance, of the reelin, glutamic acid decarboxylase, and NMDA receptor genes*) in GABAergic neurons is a consequence of promoter hypermethylation mediated by the overexpression of DNA methyltransferase in schizophrenic patients [129-131].

However, production of oxygen, free radicals and glutathione (GSH) influences gene expression and chromatin structure [132]. GSH is the key thiol antioxidant and redox buffer in cells and is present in high concentrations (*in millimolar* *concentrations*) in the cytosol, nuclei, and mitochondria [133]. When GSH production increases, it affects DNA and histone methylation by limiting the availability of *S-*adenosylmethionine (*a general co-substrate involved in methyl group transfers*), which is a cofactor utilized during epigenetic control of gene expression by DNA and histone methyltransferases. Although a great number of experiments have provided evidence that reactive species act as fundamental signals during physiological (*pathophysiological*) processes in cells and the brain, stressful events can produce redox imbalances and unregulated free radical production in neurons, which cause DNA injury and can influence gene expression by affecting DNA (*and histone*) methylation. In addition, excess free radical production perturbs the GSH antioxidant and redox buffer system. In brief, redox control also plays an essential role in epigenetic mechanisms of schizophrenia.

## REDOX REGULATION AND NEUROLEPTIC COMPOUNDS

Neuroleptics are antipsychotic compounds used in the treatment of mental illnesses. Both first-generation antipsychotics (also called conventional or typical antipsychotics, classified according to their chemical structure, (for instance, chlorpromazine, haloperidol, perphenazine, etc.) and second-generation antipsychotics (also called atypical antipsychotics, classified according to their pharmacological properties, for instance, risperidone, quetiapine, clozapine, olanzapine, etc.) are principally dopamine antagonists that are effective in treating the positive symptoms of psychosis. More recent research is questioning the view that second generation antipsychotics are superior to first generation typical anti-psychotics [134]. However, there is the lack of effectiveness of typical and atypical antipsychotic drugs on cognitive and emotional impairments in schizophrenia [135-137]. Recently, there is an evolving picture about schizophrenia moving from dopamine to glutamatergic-centered hypothesis [138, 139].

However, because various antipsychotics exert diverse effects on neurotransmitter processes, and many neurotransmitters (*catecholamines and serotonin*) have direct free radical scavenging property, and redox modulation is an essential system in the regulation of the NMDA receptors, it means that antipsychotics basically effects on redox-linked neurotransmitter communication.

In addition, antipsychotics have not only therapeutic benefits, but they also produce significant side effects such as weight gain, lowered life expectancy, agranulocytosis (*lowered white blood cell count that includes neutrophils, basophils, and eosinophils*), tarditive dyskinesia *(repetitive, involuntary movements, such as grimacing, tongue protrusion, lip smacking, rapid eye blinking, etc.*), diabetes,http://en.wikipedia.org/wiki/Diabetes sexual dysfunction, etc. [140-142]. Neuroleptics can alter the blood-brain barrier and increase iron transport into the brain from peripheral stores [143, 144], which can be linked to the extrapyramidal motor side effects and to the pathophysiology of tardive dyskinesia. However, dopaminergic function is dependent on redox-active iron metabolism [143]. The increase of redox-active iron levels in the brain may cause cognitive impairment.

Side effects of antipsychotics are also linked to the impairment of normal mitochondrial processes (*functional and ultrastructural* *mitochondrial malfunctions*) [145, 146]. Recently, Casademont *et al.* [147] concluded that both typical and atypical antipsychotics inhibit the mitochondrial electron transport chain. For instance, antipsychotic valproic acid induces homocysteine elevation (*elevation of homocysteine (a thiol-containing amino acid that is formed when methionine is converted to cysteine) in the plasma is correlated with complex diseases, including cardiovascular and neurodegenerative diseases*) that influences the redox homeostasis and can contribute to neuronal degeneration and mitochondrial dysfunctions due to its excitotoxic (*chronic dysregulation of the intracellular Ca^2+^ homeostasis*) properties [148, 149]. Homocysteine decreases intracellular glutathione peroxidase activity and alters mitochondrial gene expression, structure, and function [150]. 

However, mitochondria are key determinants of the excitability and viability of neurons and operate as metabolic and redox hubs. In brief, both useful and harmful side effects of antipsychotics are also linked to the redox-dependent neurotransmitter and redox-dependent neurobiochemical processes.

## SUMMARY AND CONCLUSIONS: LOCAL PAROXYSM AND REDOX PROCESSES IN SCHIZOPHRENIA

Burke suggested [151] that schizophrenic visual hallucinations may be due to deafferentation and dysintegration of definite visual structures that induce an increase in the excitability of deafferented neurons. This deafferentation is associated with an increase in spontaneous activity and synchronization of nerve discharges. Thus, hallucination may be considered as a local paroxysm in some visual structures.

Stressful events can lead to redox imbalances and inflammatory processes in the brain. This atypical redox regulation induces anomalies, including mitochondrial dysfunctions. Although gene expression changes are usually attributed to mutations, epigenetic processes also play an essential role in controlling gene expression. Thus, stressful incidents produce metabolic defects that affect epigenetic enzymes and cause uncontrolled overproduction of reactive species that alter DNA methylation and histone modifications. Finally, stressful- driven processes can lead to the regression of synapses and deafferentiation of the brain circuits during neurodevelopment. This regression entails the loss of synaptic spines, which is under the control of the activity of NMDA receptors on the spines. It is possible that during neurodevelopment in prenatal and early life, genetic and environmental stress factors cause atypical formation of separated local (*small*) neuron groups.

These partially isolated local neuron groups may work in a random manner as closed-loop synchronized units with increased excitability and produce local paroxysms. Humans’ brain circuitry is not mature until after age twenty. When the adolescent brain is swamped by stress, sex, and growth hormones, and with concomitant increases in the activity of the hypothalamic–pituitary–adrenal system, atypical separated local (*small*) neuron groups are also activated. These local neuron groups can remain partially isolated throughout life because they are separated from the perspective of information. This may explain why schizophrenia is such a complex and lifelong brain disorder.

However, most intra- and interneuronal signal processes are subject to redox control and modulation in a direct or indirect manner, as was represented in this paper. It has been accepted that glutamate receptors are the primary molecular structure controlling synaptic plasticity and memory function in the brain. Several biochemical steps of NMDA receptor activation are redox-regulated/linked processes controlled by reactive species (*free radicals*) and their derivatives. In addition, volume transmission of dopamine can capture free radicals and chelating zinc (*and other*) ions in the synaptic cleft and may have an important role in retrograde signaling *via* metabotropic GluR mechanisms in a G-protein-mediated manner.

Moreover, dopamine can reversibly regulate (*net inhibitory effect*) mitochondrial motility. Thus, excess dopamine can produce dysfunctions of mitochondrial distribution (i.e., *perturbation of mitochondrial fusion and fission processes)* and metabolic functions as has also been suggested for schizophrenia. Because neuronal activity and energy metabolism are direct coupled mechanisms, and regions high in neuronal activity - particularly the glutamatergic ones - have high levels of mitochondrial activity, excess dopamine can perturb neuronal activity *via* the expression of mitochondrial networks and glutamatergic NMDA receptors.

Additionally, although dopamine *o*quinone can rapidly recover to dopamine, excess dopamine production - in the context of insufficient antioxidants - results in formation of neurotoxic *o*semiquinone free radicals from *o*quinones. These free radicals also cause malfunctions of synaptic processes.

Phosphorylation of NMDAR subunits by protein kinases is also free radical associated. In addition, redox-mediated activation of NMDA receptors induces a series of further redox-associated free radical signaling processes, such as NADPH oxidase activity, neuronal nitric oxide synthase (nNOS) activity, mitochondrial enzyme activity, induction of the arachidonic acid cascade, phospholipase A, and prostaglandin H (PGH) synthase. Furthermore, research has shown that serotonin can modulate dopaminergic functions.

Since dopamine can exert a net inhibitory effect on mitochondrial movement (*mitochondria* *are key determinants of the excitability and viability of neurons and act as metabolic hubs*) and induce overproduction of H_2_O_2_ and superoxides *via* monoamine oxidase, which inhibits mitochondrial respiration, this restriction of mitochondrial movement (*fusion, fission, redistribution*) and respiration can constrain the ongoing neuronal information processes and cause closed-loop synchronized activity in local neuron groups that can produce local paroxysms. In addition, because the dopamine primordially performs volume transmission (*and most dopamine receptors are positioned at extrasynaptic sites*), excess dopamine production in local deafferented neuron areas can have an especially important regulatory effect on the release of various neurotransmitters (*and their receptors*) and on classic synaptic communication.

Because the majority of dopamine (*monoaminergic*) varicosities create nonsynaptic contact that enables the release of transmitters directly into the extrasynaptic space, catecholamines and serotonin have free radical scavengers, and the effect of nonsynaptic volume neurotransmission (*paracrine or diffusion neurotransmission*) may be not as exact as classical communication by the synaptic mechanism, it is also very important to approach complex causal mechanisms of schizophrenia from a nonsynaptic and redox point of view. 

## Figures and Tables

**Fig. (1) Dopamine volume neurotransmission and some redox processes during activation of NMDA, AMPA and MGLU receptors.**
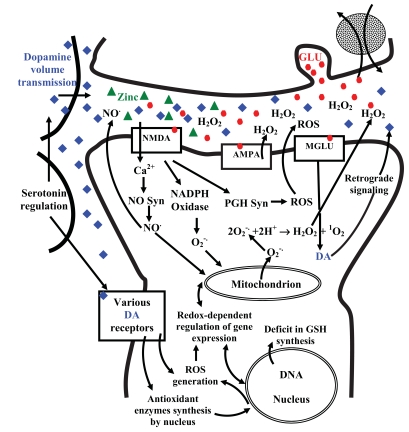
DA, dopamine; NO, nitric oxide; NO Syn, nitric oxide synthetase; MGLU, metabotropic glutamate receptor; NMDA, N-methyl-d-aspartate ionotropic glutamate receptor; AMPA, α-amino-3-hydroxy-5-methyl-4-isoxazolepropionic acid ionotropic glutamate receptor; PGH Syn, prostaglandin H synthetase; ROS, reactive oxygen species; GLU, glutamate; H_2_O_2_, hydrogen peroxide; O_2_^-.^ superoxide anion; ^1^O_2_ singlet oxygen; GSH, Glutathione.
